# Influence of pyrolysis temperature on the physicochemical properties of biochars obtained from herbaceous and woody plants

**DOI:** 10.1186/s40643-022-00618-z

**Published:** 2022-12-22

**Authors:** Panfeng Tu, Guanlin Zhang, Guoqiang Wei, Juan Li, Yongquan Li, Lifang Deng, Haoran Yuan

**Affiliations:** 1 Zhongkai University of Agriculture and Engineering, Guangzhou, 510225 People’s Republic of China; 2 Guangzhou Institute of Energy Conversion, Chinese Academy of Sciences, Guangzhou, 510640 China; 3 Institute of Biomass Engineering, South China Agricultural University, Guangzhou, 510642 People’s Republic of China

**Keywords:** Biochar, Pyrolysis temperature, Herbaceous plants, Woody plants

## Abstract

This work aimed to investigate the effect of pyrolysis temperature on the yield and properties of biochars synthesized from herbaceous and woody plants. Four typical materials, including two herbaceous plants (rice straw, corn straw) and two woody plants (*camellia oleifera* shells, garden waste), were used in the experiments under five operating temperatures (from 300 °C to 700 °C, with an interval of 100 °C). The results showed biochar derived from herbaceous plants had a significantly higher pH (from 7.68 to 11.29 for RS), electrical conductivity (EC, from 6.5 Ms cm^−1^ to 13.2 mS cm^−1^ for RS), cation exchange conductivity (CEC, from 27.81 cmol kg^−1^ to 21.69 cmol kg^−1^ for RS), and ash content (from 21.79% to 32.71% for RS) than the biochar from woody plants, but the volatile matter (VM, from 42.23% to 11.77% for OT) and specific surface area (BET, from 2.88 m^2^ g^−1^ to 301.67 m^2^ g^−1^ for OT) in the woody plant-derived biochar were higher. Except for CEC and VM, all the previously referred physicochemical characteristics in the as-prepared biochars increased with the increasing pyrolysis temperature, the H/C and O/C values of herbaceous and woody plant-derived biochar were lower than 0.9 and 0.3, respectively, confirming their potential as the material for carbon sequestration. The results revealed that biochar made from herbaceous plants was more suitable for acidic soil amendments. In contrast, woody plant-derived biochar were recommended to remove heavy metals in environmental remediation and water treatment.

**Graphical Abstract:**

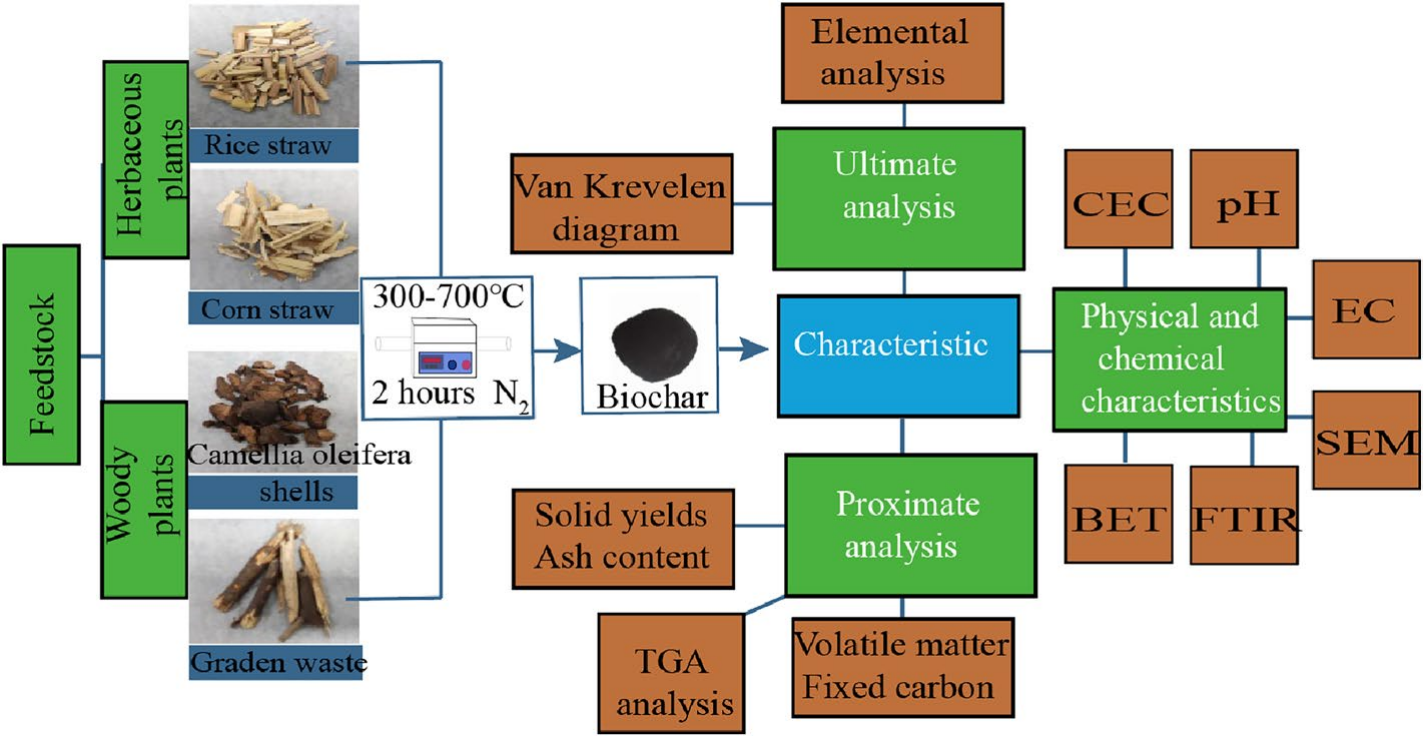

**Supplementary Information:**

The online version contains supplementary material available at 10.1186/s40643-022-00618-z.

## Introduction

In China, a large amount of agricultural and forestry wastes are produced every year, which poses a great challenge to clean treatment and utilization of resources (Chernyaeva et al. 2017; Wei et al. 2020). For example, the residual amount of corn straw in China is as high as 354.49 million tons, the rice straw is about 195.8 million tons (Ji 2015), and the garden waste in China reached 40 million tons in 2019 (Liu et al. 2020). If those solid wastes are not properly used or treated, they may cause environmental problems. Moreover, the physicochemical property of agricultural and forestry residues were different from feedstocks to feedstocks (Cai et al. 2021; Liao et al. 2018). Thus, it is necessary to conduct systematic studies on different types of raw materials.

Agricultural and forestry wastes are characterized by a large amount and abundance of organic materials, such as cellulose, hemicellulose, and lignin. For a long time, they are mainly disposed of landfill, composting, and used in incineration for energy generation, fillers in polymer composites, and biopolymers development (Kaur et al. 2021). Nowadays, more than 40% of agricultural and forestry wastes are directly incinerated in China (Cheng et al. 2011; Clare et al. 2015), which wastes resources, causes air pollution, and even endangers human health (Qu et al. 2012). Pyrolysis is a thermal treatment method with bio-oil, biochar, and combustible gas as the main products conducted under anaerobic or anoxic conditions at 300 ~ 800 °C. It is considered one of the alternative methods for thermochemical treatment of agricultural, forestry wastes and industrial waste, etc. (Lehmann et al. 2006), and depending on the pyrolysis temperature, about 20–50% (mass) of the carbon-containing material can be changed into biochar (Al-Wabel et al. 2013). Biochar is an alkaline solid with high porosity, large specific surface area, rich in aromatic carbon and surface functional groups (such as -OH, -COOH, C = O, and C − O), which can be derived from the pyrolysis of plant, animal, or microorganisms, etc.

Based on its unique chemical and physical properties, biochar was extensively used as solid fuel, gasification catalyst (Yao et al. 2016), absorbents of heavy metals (Abdelhadi et al. 2017), carbon sequestration, and soil amendments (Zimmerman 2010). When used as soil amendments, biochar could increase soil pH, porosity, water retention capacity, C/N ratio, soil total/organic carbon storage, soil total N and P, and soil cation exchange capacity. In addition, biochar amendment promotes the uptake of N and P fertilizers, root growth, and crop production to improve acidic soils and mitigate greenhouse gas emissions (Ginebra et al. 2022; Zhang et al. 2020a). Moreover, various researchers studied the effect of pyrolysis temperature and feedstock type on biochar properties. It was found that different feedstocks treated under varying pyrolysis temperatures may remarkably affect the chemical and physical properties of the obtained biochar (Hassan et al. 2020; Ortiz et al. 2020). However, there is a lack of knowledge on clarifying the biochar application scenarios according to feedstocks types and pyrolysis temperatures.

Commonly, plants can be divided into woody and herbaceous plants, and they are different in the content of three major components (lignin, cellulose, and hemicellulose). Woody plants such as fir, pine, aspen, beech, etc. were with relatively higher lignin content, but herbaceous plants such as rice straw, wheat straw, corn straw, cotton straw, etc. were higher in cellulose and hemicellulose content. Therefore, the biomass from herbaceous plants have a lower thermal stability and their macromolecules undergo cracking more easily, yielding more bio-oil and non-condensable gases than that from woody plants (Burhenne et al. 2013). Moreover, different pyrolysis mechanisms and products are presented as lignin, cellulose, and hemicellulose with unique structures and different functional groups. For example, the high content of benzene rings and methoxyl groups in lignin are the moieties cause for explaining the high char yield (Collard et al. 2014; Wang et al. 2015a), and hemicellulose in the structure of xylan is more likely to yield char than that in monosaccharides (Wang et al. 2013). In addition, the pyrolysis behavior of whole biomass and the pyrolysis product distribution is closely related to the proportion and the form of major components (Wang et al. 2017). Biochar is the solid residue after pyrolysis and favors a formation mechanism of intra-, intermolecular rearrangement and cyclization reactions, its chemical and physical properties were closely related to the composition of the major components in feedstocks, and the properties discrepancy in biochar leading to a diverse application scenario (Collard et al. 2012). Therefore, it is necessary to classify and summarize the physicochemical properties of herbaceous and woody plant-derived biochar to provide general guidance for its application.

To find out the influence of feedstocks types and pyrolysis temperatures on the physicochemical properties of the as-prepared biochars, and then clarify the biochar application scenarios accordingly, the yield and four different parts characteristic of the biochar that prepared from two herbaceous plants (rice straw, corn straw) and two woody plants (camellia oleifera shells, garden waste) under five operating temperatures (300 ºC, 400 ºC, 500 ºC, 600 ºC, and 700 ºC) were analyzed in this study. It is hoped to compare the herbaceous plant-derived biochar with woody plant-derived biochar to evaluate their potential application value.

## Materials and methods

### Materials

In this study, two woody plants (*camellia oleifera* shells and garden waste) and two herbaceous plants (rice straw and corn straw) were selected, considering their vast quantity and lacking proper disposal techniques. The rice straw and corn straw were collected from the farmland in Guangzhou, *Camellia oleifera* shells was obtained from a *C. oleifera* oil factory in Meizhou, and garden waste was mainly the branches of *Banian* from Guangzhou garden rubbish.

### Preparation of biochar

Rice straw, corn straw, *camellia oleifera* shells, and garden waste were dried at 105 ºC for 12 h, and cut into small pieces (about 2 ~ 3 cm). Then, the small pieces were pyrolyzed in a horizontal quartz reactor at the temperature range of 300 ºC–700 ºC for 120 min with a heating rate of about 10 ºC/min under the shielding gas of N_2_. Subsequently, the as-prepared biochar particles were ground by mortar and pestle and sieved to obtain particles with a diameter smaller than 0.5 mm. The as-prepared biochars with rice straw, corn straw, *camellia oleifera* shells, and garden waste as feedstocks are labeled as RS, CS, OT, and GW, respectively. The abbreviations and corresponding pyrolysis temperatures of the as-prepared biochar are presented in Additional file 1: Table S2.

### Biochar characterization

#### Elemental analysis

An elemental analyzer (EA3000, Italy) was used to analyze the elemental composition (C, N, and H) of the as-prepared biochar. An approximately 3–5 mg sample was placed in pressed tin boats, extrusion moulding, then an elemental analyzer was used to analyze the content of C, H, and N directly. The content of O is obtained by subtraction. All samples were detected in triplicate.

$${\text{O }}\left( \% \right) = {1}00 \, \left( \% \right) - {\text{C }}\left( \% \right) -{\text{H }}\left( \% \right) - {\text{N }}\left( \% \right) - {\text{Ash }}(\% )$$

#### Proximate analysis

American Society for Materials and Testing (ASTM D2866–11) was used to perform the proximate analysis (Yuan et al. 2014), and volatile matter content (VM) was conducted by ASTM D5832-98. Briefly, volatile matter was determined as the weight loss after heating the char in a covered crucible to 950 ℃and holding for 7 min, cooled to room temperature and weigh. Moisture was determined as the weight loss after heating the char in an open crucible to 107 ℃ and holding at this temperature until sample weight stabilized. The ash content was determined by placing the as-prepared biochar in a muffle furnace and heated to 950 ºC with a heating rate of about 10 ºC/min, holding for 6 h until the constant weight was obtained. The fixed carbon (FC) (wt%) content was calculated as

$${\text{FC }}\% \, = { 1}00 \, \% \, - {\text{Ash }}\% \, - {\text{VM }}\%$$

The following formula was conducted to calculate the yield of biochar (Nocentini et al. 2010):

$${\text{Yield }}\left( {{\text{wt}}. \, \% } \right) \, = {\text{ biochar weight }}\left( {\text{g}} \right) \, /{\text{ biomass weight }}\left( {\text{g}} \right) \, *{1}00\%$$

Thermo gravimetric analyzer was used to carry out the thermogravimetric analysis (TGA), with a heating rate of about 10 ºC/min, with Ar as protective gas and the pyrolysis of the material at 40–900 ºC was observed in a static-air atmosphere with 10 mg samples at a time.

#### Agronomical properties

Electrical conductivity (EC) and pH were detected by conductivity and pH meter. Biochar (1 g) and deionized water were mixed at a rate of 1:20, and the pH of the solution was measured as soon as after shaking for 30 min but EC after 24 h (Al-Wabel et al. 2013). The method of saturation with NH_4_OAC (1.0 mol/L) and Phonate method using colorimetry was conducted to test the cation exchange capacity (CEC) (Song et al. 2012). Micromeritics Sortometer—(ASAP 2050, America) was performed to detect the specific surface area (BET) by N_2_ adsorption isotherms at 77 K.

#### FTIR and SEM analysis

Identification of functional groups by molecular absorption spectrophotometry in the infrared region with Fourier transform (FTIR) (Jasco FTIR 4100, Japan), biochar samples and potassium bromide (KBr) were mixed at a ratio of 1% (w/w), using spectra obtained with 32 scans, with wavenumber from 4000 to 400 cm^−1^ and resolution of 4 cm^−1^. Scanning Electron Microscopy (SEM, Carl Zeiss-SUPRA 55, Germany) analysis was used to observe the microscopic structures, and the biochar samples were fixed on black conductive adhesive and coated with Pt to enhance the conductivity of the samples. Images were captured at 4000 magnification and operated in high vacuum mode with 2 kV accelerating voltage.

### Statistical analysis

Analyses of variances (ANOVA) was performed by Statistix version 8 (Statistix 8, Analystical, Tallahassee, FL, USA). Comparisons of means among different P treatments were made according to the least significant difference (LSD) at the 5% probability level.

## Results and discussion

### Ultimate analysis

#### Elemental analysis

The elemental analysis result is presented in Table 1. As can be seen, the C contents in the feedstocks were lower, but H and O contents were much higher than that in the as-prepared biochars. It is worth noting that the N contents in biochar were higher than that in feedstocks when the pyrolysis temperature were lower than 500 °C, but a reversed result can be seen when pyrolysis temperature were higher than 500 °C. The total C content in herbaceous and woody plant-derived biochar all increased with the temperature increase. At the same time, the H and O decreased, which can be attributed to the cracking of weak bonds in the biochar (Sun et al. 2014). The proximate analysis and chemical compositions of the feedstocks are presented in Additional file 1: Table S1 and S3, woody plants (*camellia oleifera* shells and garden waste) contain higher lignin content and volatile matter than herbaceous plants (rice straw and corn straw); however, the ash in herbaceous plants was higher than that in woody plants. Due to the higher lignin contained in woody plants (Additional file 1: Table S1) and the easily and thoroughly breakdown of herbaceous plants, the total carbon content in woody plant-derived biochar (OT and GW) was up to about 65.9 ~ 83.4%, much higher than that of herbaceous plant-derived biochar (RS and CS, about 58.4 ~ 78.7%). N contents in the as-prepared materials ranged from 0.10% to 1.71%, which decreased with temperature increase. In addition, attributing to the higher N content in herbaceous plants, N content in the herbaceous plant-derived biochar was up to 1.71%, higher than that in woody plant-derived biochar (Wang et al. 2015b), hinting its application in oxygen reduction reaction and CO_2_ reduction reaction (Deng et al. 2017; Yuan et al. 2020).

**Table 1:** Different pyrolysis temperatures and elemental content of biochar

Sample	Elemental analysis (Dry basis)				Molecular ratio	
	C (%)	H (%)	N (%)	O (%)	H/C	O/C
Rice straw	39.81 ± 1.12	5.74 ± 0.08	0.64 ± 0.04	42.56 ± 0.37	1.73 ± 0.012	0.86 ± 0.016
RS3	58.36 ± 1.25	3.51 ± 0.07	0.89 ± 0.01	15.45 ± 0.68	0.72 ± 0.013	0.20 ± 0.013
RS4	60.38 ± 1.32	3.01 ± 0.06	0.73 ± 0.02	11.95 ± 0.54	0.60 ± 0.012	0.15 ± 0.012
RS5	61.98 ± 1.10	2.32 ± 0.04	0.62 ± 0.03	6.50 ± 0.12	0.45 ± 0.014	0.08 ± 0.011
RS6	62.44 ± 1.02	1.90 ± 0.03	0.40 ± 0.01	5.39 ± 0.21	0.37 ± 0.011	0.07 ± 0.009
RS7	64.34 ± 1.43	1.77 ± 0.03	0.30 ± 0.02	0.88 ± 0.06	0.33 ± 0.010	0.01 ± 0.011
Corn straw	41.11 ± 1.25	5.12 ± 0.06	0.97 ± 0.02	48.35 ± 0.51	1.49 ± 0.016	0.88 ± 0.014
CS3	67.88 ± 0.89	3.79 ± 0.04	1.71 ± 0.01	14.73 ± 0.39	0.67 ± 0.012	0.16 ± 0.012
CS4	71.56 ± 1.62	3.52 ± 0.09	1.64 ± 0.04	10.57 ± 0.32	0.59 ± 0.013	0.11 ± 0.011
CS5	73.80 ± 1.14	2.70 ± 0.08	1.60 ± 0.03	8.52 ± 0.42	0.44 ± 0.011	0.09 ± 0.012
CS6	76.70 ± 1.92	2.14 ± 0.05	0.90 ± 0.01	5.70 ± 0.32	0.34 ± 0.012	0.06 ± 0.012
CS7	78.71 ± 1.82	2.07 ± 0.04	0.63 ± 0.03	2.91 ± 0.36	0.32 ± 0.013	0.03 ± 0.008
Camellia oleifera shells	47.53 ± 1.53	5.96 ± 0.04	0.41 ± 0.02	41.84 ± 0.69	1.50 ± 0.017	0.65 ± 0.011
OT3	65.93 ± 1.39	4.57 ± 0.05	0.52 ± 0.05	19.49 ± 0.57	0.83 ± 0.010	0.22 ± 0.007
OT4	73.70 ± 1.37	3.50 ± 0.03	0.40 ± 0.02	12.13 ± 0.68	0.57 ± 0.013	0.12 ± 0.009
OT5	76.82 ± 1.26	2.74 ± 0.01	0.33 ± 0.01	9.21 ± 0.41	0.43 ± 0.010	0.09 ± 0.010
OT6	78.78 ± 1.22	2.03 ± 0.01	0.19 ± 0.01	7.48 ± 0.38	0.31 ± 0.009	0.07 ± 0.012
OT7	80.93 ± 1.09	1.43 ± 0.03	0.38 ± 0.02	5.14 ± 0.34	0.21 ± 0.008	0.05 ± 0.011
Garden waste	47.71 ± 1.04	6.23 ± 0.07	0.27 ± 0.04	44.22 ± 0.72	1.57 ± 0.015	0.70 ± 0.009
GW3	68.90 ± 1.01	4.41 ± 0.08	0.44 ± 0.02	21.98 ± 0.79	0.77 ± 0.007	0.24 ± 0.012
GW4	70.96 ± 1.62	3.52 ± 0.04	0.54 ± 0.06	19.41 ± 0.64	0.60 ± 0.008	0.21 ± 0.013
GW5	79.46 ± 1.33	2.85 ± 0.02	0.23 ± 0.03	10.52 ± 0.22	0.43 ± 0.007	0.10 ± 0.011
GW6	82.98 ± 1.45	2.05 ± 0.03	0.12 ± 0.01	7.44 ± 0.23	0.30 ± 0.006	0.07 ± 0.010
GW7	83.37 ± 1.53	1.49 ± 0.04	0.10 ± 0.01	6.31 ± 0.21	0.21 ± 0.006	0.06 ± 0.012

#### Van Krevelen diagram

O/C and H/C molecular ratios are often analyzed by the Van Krevelen diagram (Moiseenko et al. 2021). The H/C molecular ratio represents the aromaticity of biochars, a low H/C ratio indicates that the compounds have a large aromatic structure and high stability. While the O/C molecular ratio represents the degree of aging of biochars (Rodriguez et al. 2020).

**Fig. 1:**
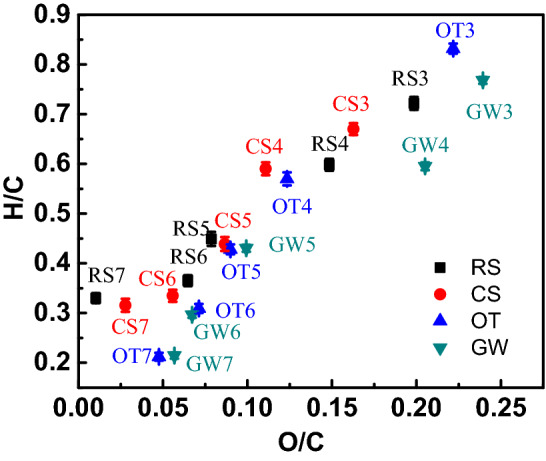
Van Krevelen graph of biochar prepared with different feedstocks

**Fig. 2:**
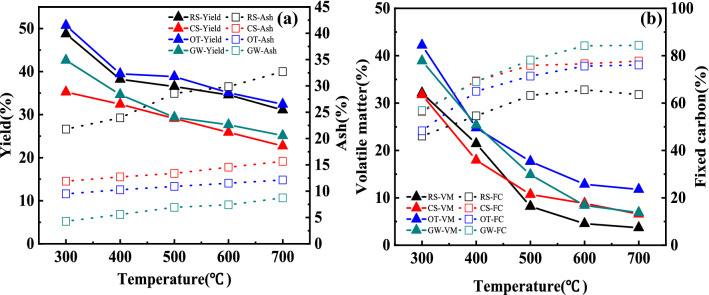
**a** Solid yields and ash content image, **b** volatile matter and fixed carbon image

As shown in Fig. 1 and Table 1, the feedstocks show the highest O/C and H/C molecular ratio. Then, due to decarboxylated dehydrogenation, the O- and H-containing functional groups disappeared gradually, O/C and H/C molecular decreased with the temperature increase (Windeatt et al. 2014). The O/C molecular ratios for the obtained biochars decreased to the range of 0.01–0.24, and the ratios in woody plant-derived biochars were higher than that in herbaceous plant-derived biochars, which indicated that woody plant-derived biochar probably had lower biological stability as it contains a large amount of aromatic organic matter (Jindo et al. 2014; Yang et al. 2007). A lower O/C molecular ratio also reflects the degradation of carbon compounds, which verified by the fact that a lower volatile matter content can be seen in higher temperature prepared biochar (Fig. 2b). Previous studies demonstrated that biochar half-life is in related to the O/C molecular ratio, a longer than 1000 year half-life is expected when biochar with an O/C molecular ratio is lower than 0.2, a half-life range of 100 ~ 1000 years is expected when biochar with the O/C molecular ratio is between 0.2 and 0.6, and the half-life may shorter than 100 years when the O/C molecular ratio is higher than 0.6. Therefore, the low O/C in this study indicated that most of the as-prepared biochar had the possibility of carbon sequestration (Elmquist et al. 2006; Wang et al. 2021).

The H/C molecular ratio in all of the as-prepared biochar was lower than 0.9, staying in the high carbonic zone of the Van Krevelen diagram, which further indicated that they had a stable construction and could be potential for carbon sequestration (Crombie et al. 2013) Moreover, because H drops more easily than O at lower temperatures, the molecules ratio of H/C drop more rapidly than O/C as the temperature increases.

### Proximate analysis

#### Solid yields and ash content

As shown in Fig. 2a and Additional file 1: Table S3, due to the less stable parts of lignin and cellulose decompose in the temperature range of 300–400 ℃, and the evaporation of water (Zhang et al. 2020b), a sharp decrease was observed in both herbaceous and woody plants at the temperature of 300–400 ℃, while a more gentle decline appeared at 400–700 ℃, with solid yields ranged from 22.71% to 50.70%, and the solid yields of OT, RS, GW and CS showed a significant difference with temperature (*P* < 0.01). Furthermore, OT got the highest solid yield, followed by RS and GW, CS presented the lowest solid yield, and a significant difference can be seen among the feedstocks (*P* < 0.01). In general, woody plants may have a higher solid yield than herbaceous plants (Ro et al. 2010), the higher ash content can explain the higher solid yield in RS.

Moreover, organism decomposition and carbonization accelerated with the raising of pyrolysis temperature, and the ash content in all biochars increased with the increase of temperature. Ash was made up of various minerals in the form of oxide, phosphate, sulfate, silicate, etc. RS presented the highest ash content range from 21.79 to 32.71%, higher than that of CS (11.89–15.68%), OT (9.49–12.12%), and GW (4.27–8.73%), which may be attributed to the higher inorganic components and mineral content in herbaceous plants (Xiao et al. 2014). As can be seen from Additional file 1: Table S3, the ash content of OT, RS, GW and CS showed a significant difference with temperature (*P* < 0.01). Furthermore, RS got the highest ash content, and a significant difference can be seen among the feedstocks (*P* < 0.01). According to Rodriguez et al. (Rodriguez et al. 2020), biochar with high ash content hinted at its potential application in acidic soil improvement, so herbaceous plant-derived biochar with higher ash content and pH values may be more suitable for improving acidic soils than woody plant-derived biochar.

#### Volatile matter and fixed carbon analysis

Previous studies emphasized that feedstock, instead of pyrolysis temperature was responsible for the volatile matter content (Enders et al., 2012). However, as shown in Fig. 2b and Additional file 1: Table S3, the volatile matter of RS, CS, OT, and GW biochar was found to be in the range of 3.69–32.13%, 6.56–31.77%, 11.77–42.23%, and 6.88–38.91%, respectively, a significant difference can be seen in the volatile matter with temperature and feedstocks (*P* < 0.01), and biochar prepared at higher temperature presented less volatile matter than that prepared at a lower temperature. Similar findings can be found in other studies; both feedstock and pyrolysis temperature may affect the volatile matter content (Pariyar et al. 2020; Sotoudehnia et al. 2020). In addition, woody plant-derived biochar exhibited a considerably greater amount of volatile matter than herbaceous plant-derived biochar, which may be related to the higher degree of carbonization of woody plant-derived biochar and the higher lignin content but lower ash content.

Volatile matter can be easily decomposed by microorganisms, whereas fixed carbon may remain in the soil for a longer time as it is biologically and chemically recalcitrant. In this study, fixed carbon content varies with feedstock and pyrolysis temperature. The fixed carbon in all biochars ranged from 46.08 ± 0.16% to 84.39 ± 0.02%, and the highest fixed carbon was found in GW, about 56.82 ± 0.29–84.39 ± 0.02%, followed by CS (56.34 ± 0.26–77.76 ± 0.22%), OT (48.28 ± 0.33–76.11 ± 0.35%) and RS (46.08 ± 0.16–65.58 ± 0.15%), which increased with increasing temperature and showed a significant difference with temperature and feedstocks (*P* < 0.01). The relatively higher fixed carbon in woody plants derived biochar indicates its higher calorific value and potential application in carbon sequestration.

#### Thermogravimetric analysis (TGA)

The thermal stability of biochar pyrolysis in the air was analyzed by Thermo gravimetric analyzer. TGA curves in Additional file 1: Figure S1 show that pyrolysis temperature significantly affects biochar thermal stability. Biochar prepared at a high pyrolysis temperature had better stability (Sun et al. 2014). This may be related to higher carbon content, lower volatile matter, and more stable structure of materials in higher pyrolysis temperature prepared biochar.

As presented in Additional file 1: Figure S1, all the biochar curves followed a similar trend. From 25 °C (room temperature) to 200 °C, a slight weight loss can be seen due to volatilize of water from the raw material. At around 260 °C and 300 °C, a noticeable weight loss was observed in herbaceous plant-derived biochar and woody plant-derived biochar, and the weight loss temperature of biochar increased with the increasing pyrolysis temperature. The weight loss at 200–500 °C could be due to the degradation and decomposition of hemicellulose and cellulose, and carbon possibly transferred to CO_2_, CO, and CH_4_ (Sun et al. 2014). However, the decomposition of lignin began to stabilize, burnout was above 500–600 °C, and decomposition for all the samples was completed, then the curves became stable (Zhang et al. 2012).

In addition, the residual amount in herbaceous and woody-derived biochar was significantly different. Herbaceous plant-derived biochar (RS and CS) had relatively more residues left than woody plant-derived biochar (GW and OT), and the residues of the four raw materials followed the order: RS > CS > OT > GW, which can be explained by the ash content in this study.

### Physico-chemical characteristics

#### pH

**Fig. 3:**
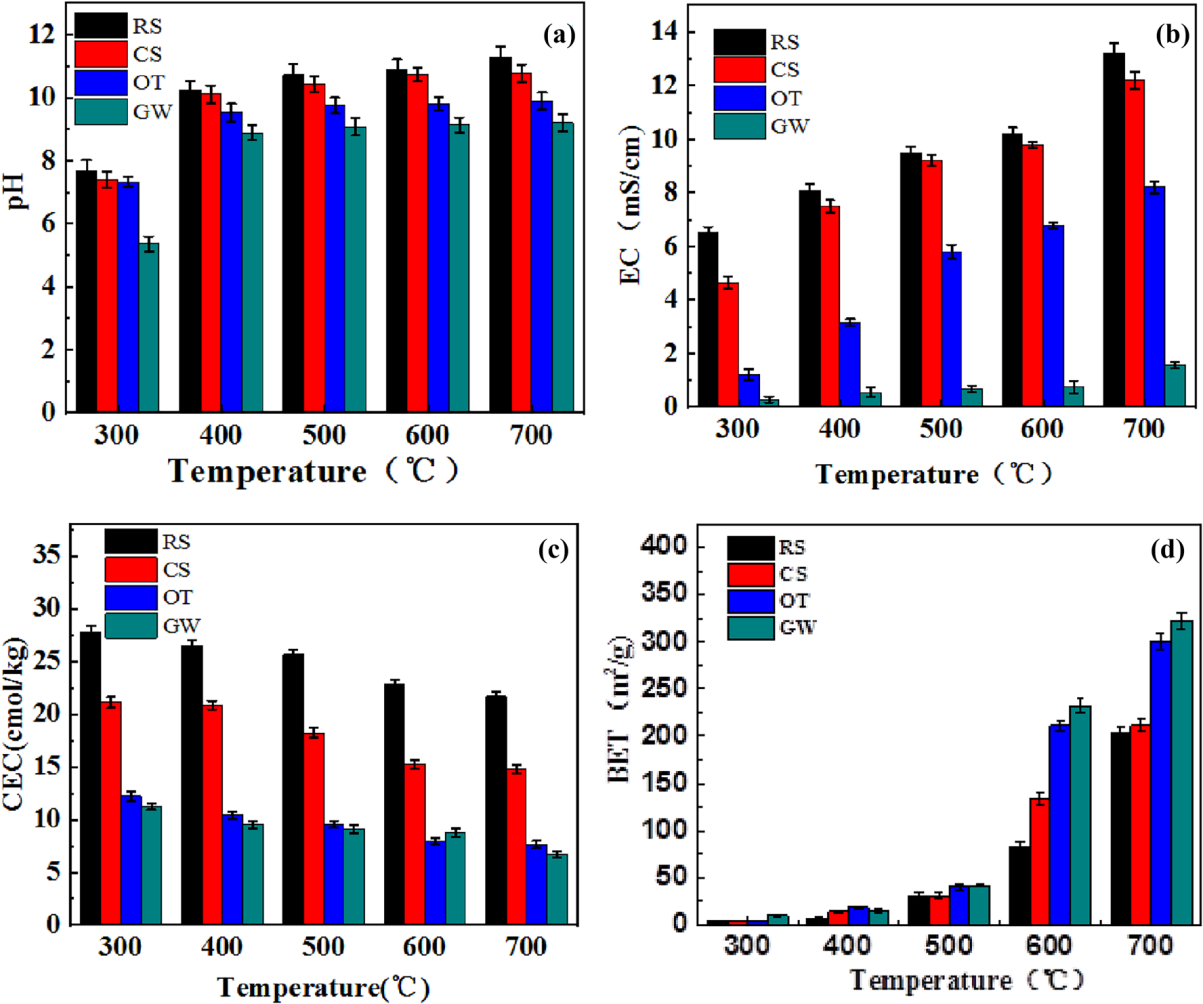
**a** pH, **b** EC value, **c** CEC, **d** BET images

Figure 3A and Additional file 1: Table S4 present the basic parameters of the biochars obtained at the temperature of 300 °C, 400 °C, 500 °C, 600 °C, and 700 °C, the pH values of the as-prepared biochars increased from 5.36 ± 0.04 to 11.29 ± 0.05 with the temperature increase, and a significant difference can be seen in pH with temperature and feedstocks (*P* < 0.01). All the biochars seem to be alkaline except GW3 (about 5.36). The result shows that both pyrolysis temperature and raw materials type were factors affecting the pH value of biochar, the pH values of the as-prepared biochars were positively correlated with pyrolysis temperature, which was also different even biochar prepared at the same temperature with other materials. The possible reasons for the increase of pH with temperature are (i) Volatile matter in biochar decreased, organic functional groups such as -OH and -COOH have a significant influence on the pH value of as-prepared biochar, but its amount decreased (Castilla-Caballero et al. 2020), (ii) The accumulation of some inorganic salts such as sodium (Na), potassium (K), magnesium (Mg), and calcium (Ca), and some carbonates (such as CaCO_3_ and MgCO_3_) and inorganic alkalis were generated at higher temperature (Yuan et al. 2011a). Besides, RS got the highest pH value, followed by CS, OT, and GW when prepared at the same pyrolysis temperature, which may be attributed to the higher ash content but lower volatile matter in RS.

According to previous studies, the application of biochar with high pH in acidic soils can improve cation exchange capacity, achieve greater neutralization and reduce greenhouse gas emissions (Ortiz et al. 2020; Yuan et al. 2011b). Therefore, as-prepared biochars, especially the herbaceous plant-derived biochar (RS and CS) with higher pH values and high ash content in this study, may be more suitable for amendment of acidic soil, which can not only reduce soil leaching, neutralize soil acidity, but also improve nutrient accessibility.

#### EC value

Normal plant growth may be obstructed as high salt content alters the soil–water balance, and soil salinity is usually expressed in terms of EC. Figure 3b and Additional file 1: Table S4 show that the EC values in RS, CS, OT, and GW biochars improved as the pyrolysis temperature increased, which is attributed to the accumulation of inorganic alkaline substances and the release of volatile matter (Azargohar et al., 2014; Narzari et al., 2017). Overall, the EC values of the samples ranged from 0.29 to 13.2 mS/cm, following the order: RS > CS > OT > GW, showed a significant difference with temperature and feedstocks (*P* < 0.01), and the highest EC value of about 13.2 mS/cm in RS7 may be attributed to its highest ash content.

Moreover, herbaceous plant-derived biochar had a higher EC value than woody plant-derived biochar, indicating that herbaceous plant-derived biochar had higher soluble salts and fewer acid functional groups (Askeland et al. 2019), which reaffirmed the availability of applying herbaceous plant-derived biochar as acidic soil amendments.

#### Cation exchange capacity

A previous study demonstrated that temperature was the main factor affecting the CEC value (Song et al. 2012). Moreover, the latent CEC values of biochars obtained from herbaceous and woody plants under different temperatures are shown in Fig. 3c and Additional file 1: Table S4. The CEC values ranged from 6.74 to 27.81 Cmol/kg, where RS3 got the highest CEC value of 27.81 Cmol/kg, the higher CEC value in herbaceous-derived biochar was the result of the presence of exchangeable Ca^2+^ (Hailegnaw et al. 2019), and a significant difference can be seen in the CEC values with temperature and feedstocks (*P* < 0.01). In addition, surface organic functional biochar groups are responsible for CEC function, and the CEC values of biochar with four raw materials are negatively correlated with the increase in pyrolysis temperature (Song et al. 2012). This may be due to the loss of surface organic functional groups (Liao et al. 2018) and the oxidation reaction of C (Moreno-Castilla et al. 2004) with higher temperatures; such speculation was supported by the O/C ratio results in this study.

CEC value can be used to represent the absorption capacity of biochar to soil nutrients (Bera et al. 2018). Therefore, the application of biochar with high CEC in soil may improve plant growth by retaining nutrients such as K^+^ and NH_4_^+^ and may further prevent groundwater contamination by reducing the leaching of these nutrients (Zhang et al. 2013). Then, herbaceous-derived biochar with a higher CEC value may be more suitable as a nutrients element adsorbent.

#### BET-specific surface area analysis

Figure 3D and Additional file 1: Table S4 show the specific surface area of RS, CS, OT, and GW biochars prepared under temperatures ranging from 300 °C to 700 °C. As anticipated, the particular surface area increased with the increase in temperature (Das et al. 2016; Yang et al. 2020); GW7 got the highest specific surface area of 322.92 ± 8.11 m^2^/g, followed by the OT7 (301.67 ± 8.56 m^2^/g), CS7 (211.22 ± 6.78 m^2^/g) and RS7 (202.71 ± 7.77 m^2^/g), and a significant difference in the specific surface area can be seen among the feedstocks (*P* < 0.01). There were no significant changes in specific surface area at the pyrolysis temperature of 300 °C and 400 °C, as the branch chain carbon atoms fractured, but only micropores were formed during pyrolysis at low temperatures. However, when pyrolysis temperature keeps increasing to 500 °C, 600 °C, and 700 °C, micropores expand into mesopores, some mesopores further expand into macropores, and the volatilities of organic compounds increase, more reactants are completely carbonized (Song et al. 2012). Then, the specific surface area of almost all the as-prepared biochars improved significantly. Moreover, the N_2_ adsorption–desorption isotherms in Additional file 1: Figure S2 further confirmed our analysis. As can be seen, the adsorption branch of GW3 and GW4 resemble that of a type-I isotherm in the IUPAC classification, hinted the existence of micropore. However, the weak hysteresis loop in GW5 suggests a combined I/IV-type isotherm and indicates that these materials are primarily micropores. GW6 and GW7 show typical type-IV adsorption–desorption isotherms with large hysteresis loops, suggesting the presence of mesoporous.

Previous studies demonstrated that the larger the specific surface area of a material, the more porous the structure inside it, so the specific surface area may be one of the most significant factors influencing the ability of a material to adsorb compounds (Inyang et al. 2010; Sun et al. 2014). Therefore, woody plant-derived biochar such as OT7 and GW7 with a relatively large surface area and higher CEC were probably more helpful for environmental remediation and water treatment, as they can adsorb heavy metals, modify soil structure and improve soil water holding capacity.

### FTIR and SEM analyses

#### FTIR analysis

Structure and property information of different functional groups and chemical features of the investigated substances were usually obtained by FTIR spectral analysis. Additional file 1: Figure S3 and Table 2 show the FTIR spectra of biochars prepared from different feedstocks at different pyrolysis temperatures. For all the as-prepared biochars, the spectral peaks observed between 3600 cm^−1^ to 3000 cm^−1^ were assigned to the vibration of the -OH bonds in phenols, carboxylic acids, and alcohols. Peaks presented between 2960 and 2800 cm^−1^ were related to C–H groups, figuring the aliphatic group, such as methylene (CH_2_) and methyl (CH_3_). The range between 2300 to 2200 cm^−1^ may be attributed to triple bonding, for example, nitriles (C≡N) and alkynes (C≡C) (Larkin 2011). The peaks at approximately 1650 to 1550 cm^−1^ were probably because of the vibration of the C = O and C = C, representing the existence of aromatic hydrocarbons, alkenes, ketones, aldehydes, and carboxylic acid (Haeldermans et al. 2019), or amide group (N–H) either (Rodriguez et al. 2020). The aliphatic -CH_3_ stretching and C–O groups were found in ranges between 1475 and 1350 cm^−1^ and 1020 to 1066 cm^−1^, and the latter group demonstrated the presence of ethers and esters.

**Table 2:** FTIR spectra and their corresponding functional group

Wavenumbers (cm^−1^)	Band assignments
3000–3600	–OH stretching (Haeldermans et al. 2019)
2800–2960	Asymmetric C–H stretching (Ma et al. 2022)
2200–2300	Triple bonding such as nitriles (C≡N) and alkynes (C≡C) (Larkin 2011)
1550–1650	Aromatic ring C=C; C=O stretching, or amide group (N–H) (Haeldermans et al. 2019; Rodriguez et al. 2020)
1350–1445	CH_2_ units in biopolymers (Dong et al. 2016)
1020–1066	Stretching of ring breathing C–O (Liu et al. 2015)

The FTIR spectra demonstrated that the pyrolysis temperature presented the same effect on biochars with different feedstocks. Peaks for –OH and C=O in RS, CS, OT and GW became weaker with the temperature increase, indicating the reduction of carbonyl and hydroxyl. Herbaceous and woody plant-derived biochar had little difference in functional group species as all of them had –OH, C=O, C–H, and other functional groups. However, the peaks of C–O, C–H, and –OH in woody plant-derived biochars were weaker than that in herbaceous plant-derived biochar. Moreover, the functional group of –OH and –COOH on the biochar surface indicated its potential for heavy metal adsorption, the existence of such functional group in all of the as-prepared biochar hinted at their application in heavy-metal removal in the wastewater.

#### SEM analysis

The images of woody plants and herbaceous plants' raw materials and SEM images of RS, CS, OT, and GW prepared at the temperature of 300–700 °C are presented in Additional file 1: Figure S4. All of the biochars prepared at 300 °C and 400 °C had a smooth surface, and pores were observable in biochars of OT5, OT6, GW5, and GW6, while RS5, RS6, CS5, and CS6 biochars had smaller particle sizes. The images further depicted that porous structures were observed in all of the biochars obtained at the temperature of 700 °C, which may be attributed to the organic compounds volatilization and volatile matter release (Gupta et al. 2018). Obviously, the pores number in RS, CS, OT, and GW increased with the increase in temperature, and CS7 was found to have a more porous structure, followed by GW7, RS7, and OT7, which was supported by the BET surface analysis.

## Conclusions

This study shows that the yield and properties of the biochars obtained from herbaceous and woody plants presents much difference when prepared at different pyrolysis temperatures. It was observed that as the pyrolysis temperature increased from 300 °C to 700 °C, the H/C and O/C molecular ratio, solid yield (from 50.70% to 32.41% for OT, and from 48.75% to 31.08% for RS), cation exchange capacity (from 12.24 cmol kg^−1^ to 7.65 cmol kg^−1^ for OT, and from 27.81 cmol kg^−1^ to 21.69 cmol kg^−1^ for RS), and volatile matter (from 42.23% to 11.77% for OT, and from 32.13% to 3.69 for RS) in the as-prepared biochars decreased, while the pH (from 7.32 to 9.89 for OT, and from 7.68 to 11.29 for RS), specific surface area (from 2.88 m^2^ g^−1^ to 301.67 m^2^ g^−1^ for OT, and from 4.72 m^2^ g^−1^ to 207.71 m^2^ g^−1^ for RS), ash content (from 9.49% to 12.12% for OT, and from 21.79% to 32.71% for RS), electrical conductivity (from 1.22 mS cm^−1^ to 8.2 mS cm^−1^ for OT, and from 6.5 mS cm^−1^ to 13.2 mS cm^−1^ for RS), carbon content increased, and all of them showed a significant difference with temperature and feedstocks. Moreover, the ash content, pH, cation exchange capacity, and electrical conductivity in herbaceous plant-derived biochar seem to be higher than that of woody plant-derived biochar, making such biochar more suitable for using as an acid soil amendment, and the larger specific surface area, higher fix carbon content and higher volatile matter in woody plant-derived biochar hinted its more suitable application for heavy-metal remove in environmental remediation and water treatment.

### Supplementary Information

**Additional file 1: Table S1.** Chemical compositions of Rice straw, Corn straw, Oiltea camellia shells and Garden waste. **Table S2. **Abbreviation of the prepared biochar from different feedstocks at varying temperature. **Table S3.** Proximate analysis of the obtained biochars. **Table S4. **Physicochemical properties of the obtained biochars. **Fig. S1.** TG images of different temperatures of the (a) RS, (b) CS, (c) OT and (d) GW. **Fig. S2.** N_2_ adsorption–desorption isotherms of GW3, GW4, GW5, GW6, and GW7. **Fig. S3.** FTIR spectra of (a) RS, (b) CS, (c) OT and (d) GW biochars prepared at 300℃ to 700℃. **Fig. S4.** Photos of woody plants and herbaceous plants raw materials (a), SEM images of RS, CS, OT and GW prepared at (b) 300, (c) 400, (d) 500, (e) 600, (f) 700℃, respectively.

## Data Availability

All data supporting this article’s conclusion are available.
