# Supplementary material for: Influence of pyrolysis temperature on the physicochemical properties of biochars obtained from herbaceous and woody plants
Source: Bioresour Bioprocess. 2022 Dec 22;9(1):131. doi: 10.1186/s40643-022-00618-z (PMC10991468; doi:10.1186/s40643-022-00618-z)
Supplement: Supplementary file 1 — Additional file 1: Table S1. Chemical compositions of Rice straw, Corn straw, Oiltea camellia shells and Garden waste. Table S2. Abbreviation of the prepared biochar from different feedstocks at varying temperature. Table S3. Proximate analysis of the obtained biochars. Table S4. Physicochemical properties of the obtained biochars. Fig. S1. TG images of different temperatures of the (a) RS, (b) CS, (c) OT and (d) GW. Fig. S2. N2 adsorption–desorption isotherms of GW3, GW4, GW5, GW6, and GW7. Fig. S3. FTIR spectra of (a) RS, (b) CS, (c) OT and (d) GW biochars prepared at 300℃ to 700℃. Fig. S4. Photos of woody plants and herbaceous plants raw materials (a), SEM images of RS, CS, OT and GW prepared at (b) 300, (c) 400, (d) 500, (e) 600, (f) 700℃, respectively. [file 40643_2022_618_MOESM1_ESM.docx]

**Supporting materials**

**Influence of pyrolysis temperature on the physicochemical properties of biochars obtained from herbaceous and woody plants**

Panfeng Tu ^a^, Guanlin Zhang ^a, b,1^, Guoqiang Wei ^c^, Juan Li ^a^, Yongquan Li ^a^, Lifang Deng ^c*^, Haoran Yuan ^b,c^

^a^ Zhongkai University of Agriculture and Engineering, Guangzhou 510225, P.R. China.

^b^ Guangzhou Institute of Energy Conversion, Chinese Academy of Sciences, Guangzhou 510640, China.

^c^ Institute of Biomass Engineering, South China Agricultural University, Guangzhou 510642, P.R. China.

**^*^ Corresponding author:** Lifang Deng, Email: [nannandeng@163.com](mailto:nannandeng@163.com)

^1^ Co-first author

**TableS1** Chemical compositions of Rice straw, Corn straw, Oiltea camellia shells and Garden waste

| Feedstocks | Chemical compositions in this study | | |  | Chemical compositions in literatures | | |  |
| --- | --- | --- | --- | --- | --- | --- | --- | --- |
|  | CL (%) | HL (%) | LN (%) |  | CL (%) | HL (%) | LN (%) | Ref. |
| Rice straw | 33.55±2.52 | 18.33±1.23 | 22.13±1.77 |  | 37 | 16.5 | 13.6 | 1,2 |
| Corn straw | 27.97±1.99 | 18.14±1.54 | 20.69±1.31 |  | 42.7 | 23.2 | 17.5 | 2,3 |
| Camellia oleifera shells | 14.61±1.62 | 21.41±1.61 | 35.93±2.88 |  | - | - | - | - |
| Garden waste | 14.34±1.82 | 33.09±2.12 | 34.30±2.02 |  | 43.0 | 29.4 | 27.6 | 3 |

CL: Cellulose, HL: Hemicellulose, LN: Lignin

**References**

[1] H. Rabemanolontsoa, S. Saka, Comparative study on chemical composition of various biomass species. Rsc Adv 3 (2013) 3946–3956.

[2] T. Qu, W. Guo, L. Shen, J. Xiao, K. Zhao, Experimental study of biomass pyrolysis based on three major components: hemicellulose, cellulose, and lignin. Ind Eng Chem Res 50 (2011) 10424–10433.

[3] A. Demirbaş Thermochemical conversion of biomass to liquid products in the aqueous medium. Energy Sources 27 (2005) 1235-1243.

**TableS2** Abbreviation of the prepared biochar from different feedstock at varying temperature.

| abbreviation | | details |
| --- | --- | --- |
| RS | RS3 | Rice straw biochar prepared at 300℃ |
|  | RS4 | Rice straw biochar prepared at 400℃ |
|  | RS5 | Rice straw biochar prepared at 500℃ |
|  | RS6 | Rice straw biochar prepared at 600℃ |
|  | RS7 | Rice straw biochar prepared at 700℃ |
| CS | CS3 | Corn straw biochar prepared at 300℃ |
|  | CS4 | Corn straw biochar prepared at 400℃ |
|  | CS5 | Corn straw biochar prepared at 500℃ |
|  | CS6 | Corn straw biochar prepared at 600℃ |
|  | CS7 | Corn straw biochar prepared at 700℃ |
| OT | OT3 | Camellia oleifera shells biochar prepared at 300℃ |
|  | OT4 | Camellia oleifera shells biochar prepared at 400℃ |
|  | OT5 | Camellia oleifera shells biochar prepared at 500℃ |
|  | OT6 | Camellia oleifera shells biochar prepared at 600℃ |
|  | OT7 | Camellia oleifera shells biochar prepared at 700℃ |
| GW | GW3 | Garden waste biochar prepared at 300℃ |
|  | GW4 | Garden waste biochar prepared at 400℃ |
|  | GW5 | Garden waste biochar prepared at 500℃ |
|  | GW6 | Garden waste biochar prepared at 600℃ |
|  | GW7 | Garden waste biochar prepared at 700℃ |

**Table S3** Proximate analysis of the obtained biochars

| Samples | Yield  (%) w/w | MS  (%) w/w | Ash  (%) w/w | FC  (%) w/w | VM  (%) w/w |
| --- | --- | --- | --- | --- | --- |
| Rice straw | - | 10.77±0.28 | 11.25±0.68 | 6.31±0.32 | 71.67±0.47 |
| RS3  RS4  RS5  RS6  RS7 | 48.75±2.62a | - | 21.79±0.68d | 46.08±0.54d | 32.13±0.96a |
|  | 38.20±2.46b | - | 23.93±0.71c | 54.63±0.49c | 21.44±0.92b |
|  | 36.53±2.41b | - | 28.58±0.76b | 63.22±0.83b | 8.20±0.63c |
|  | 34.58±2.32bc | - | 29.87±0.66b | 65.58±0.60a | 4.55±0.60d |
|  | 31.08±2.05c | - | 32.71±0.73a | 63.60±0.54b | 3.69±0.63d |
| Corn straw | - | 13.33±0.26 | 4.45±0.28 | 7.36±0.28 | 74.86±0.38 |
| CS3  CS4  CS5  CS6  CS7 | 35.25±2.02a | - | 11.89±0.82d | 56.34±0.49d | 31.77±0.83a |
|  | 32.42±1.92ab | - | 12.71±0.68cd | 69.34±0.83c | 17.95±0.78b |
|  | 29.10±1.88bc | - | 13.38±0.57bc | 75.90±0.75b | 10.72±0.52c |
|  | 25.89±1.82cd | - | 14.56±0.61ab | 76.60±0.72ab | 8.84±0.77d |
|  | 22.71±1.74d | - | 15.68±0.55a | 77.76±0.68a | 6.56±0.43e |
| Camellia oleifera shells | - | 8.52±0.23 | 4.26±0.26 | 9.15±0.31 | 78.07±0.42 |
| OT3  OT4  OT5  OT6  OT7 | 50.70±2.88a | - | 9.49±0.57c | 48.28±0.73d | 42.23±0.56a |
|  | 39.48±1.85b | - | 10.27±0.74ab | 64.96±0.76c | 24.77±0.96b |
|  | 38.83±1.82b | - | 10.90±0.83ab | 71.41±0.55b | 17.69±0.97c |
|  | 35.09±1.67c | - | 11.52±0.55ab | 75.62±0.92a | 12.86±0.98d |
|  | 32.41±1.49c | - | 12.12±0.64a | 76.11±0.64a | 11.77±0.99d |
| Garden waste | - | 11.81±0.31 | 1.57±0.22 | 6.68±0.26 | 79.94±0.24 |
| GW3  GW4  GW5  GW6  GW7 | 42.62±2.02a | - | 4.27±0.37d | 56.82±0.88d | 38.91±0.90a |
|  | 34.65±2.01b | - | 5.57±0.55c | 69.06±0.81c | 25.37±0.93b |
|  | 29.38±1.98c | - | 6.94±0.62b | 78.17±0.64b | 14.89±0.99c |
|  | 27.65±1.93cd | - | 7.41±0.51b | 84.20±0.91a | 8.39±0.53d |
|  | 25.13±1.84d | - | 8.73±0.57a | 84.39±0.98a | 6.88±0.78d |
| Feedstock (F) | ** | - | ** | ** | ** |
| Temperature (T) | ** | - | ** | ** | ** |
| F×T | ns | - | ** | ** | ** |

MS: Moisture, VM: Volatiles matter, FC: Fixed carbon.

“-”: The obtained biochars were dried in oven (60℃ for 12 h) before proximate analysis, then no moisture was detectable.

FC、VM and Ash content in biochars were detected on a water-free basis (dried at 105 ℃ for 12 h).

Values followed by different lowercase letters within a column represent significant differences among the treatments in the same feedstock at P<0.05. F: Feedstock; T: Temperature; F×T: Interaction between feedstock and temperature; * and ** represent a significant difference at P<0.05 and P<0.01, respectively; ns represents a non-significant difference.

**Table S4** Physicochemical properties of the obtained biochars

| Samples | pH | EC  (mS cm^-1^) | CEC  (cmol kg^-1^) | BET  (m^2^ g^−1^) |  |
| --- | --- | --- | --- | --- | --- |
| RS3  RS4  RS5  RS6  RS7 | 7.68±0.34c | 6.5±0.22e | 27.81±0.58a | 4.72±0.11d | |
|  | 10.23±0.31b | 8.1±0.21d | 26.51±0.56b | 6.79±1.09d | |
|  | 10.73±0.33ab | 9.5±0.22c | 25.71±0.43b | 20.15±3.53c | |
|  | 10.90±0.31ab | 10.2±0.23b | 22.89±0.35c | 81.88±6.87b | |
|  | 11.29±0.32a | 13.2±0.38a | 21.69±0.42d | 207.71±7.77a | |
| CS3  CS4  CS5  CS6  CS7 | 7.40±0.26c | 4.64±0.22e | 21.11±0.51a | 2.87±0.09d | |
|  | 10.11±0.28b | 7.5±0.24d | 20.86±0.40a | 14.07±0.87c | |
|  | 10.43±0.27ab | 9.2±0.21c | 18.24±0.47b | 20.27±3.11c | |
|  | 10.74±0.22a | 9.8±0.12b | 15.24±0.38c | 133.72±5.87b | |
|  | 10.77±0.28a | 12.2±0.31a | 14.76±0.39c | 211.22±6.78a | |
| OT3  OT4  OT5  OT6  OT7 | 7.32±0.16b | 1.22±0.21e | 12.24±0.48a | 2.88±0.11e | |
|  | 9.53±0.28a | 3.16±0.12d | 10.42±0.36b | 18.13±1.06d | |
|  | 9.76±0.25a | 5.8±0.26c | 9.54±0.32c | 40.13±3.01c | |
|  | 9.82±0.22a | 6.8±0.11b | 7.99±0.29d | 211.36±5.87b | |
|  | 9.89±0.27a | 8.2±0.22a | 7.65±0.34d | 301.67±8.56a | |
| GW3  GW4  GW5  GW6  GW7 | 5.36±0.24b | 0.29±0.11c | 11.25±0.32a | 9.65±0.78d | |
|  | 8.89±0.22a | 0.56±0.18bc | 9.54±0.34b | 14.90±1.55d | |
|  | 9.08±0.28a | 0.68±0.12b | 9.11±0.38bc | 41.57±1.67c | |
|  | 9.14±0.24a | 0.75±0.23b | 8.75±0.40c | 231.85±7.33b | |
|  | 9.21±0.26a | 1.56±0.12a | 6.74±0.31d | 322.92±8.11a | |
| Feedstock (F) | ** | ** | ** | ** | |
| Temperature (T) | ** | ** | ** | ** | |
| F×T | ** | ** | ** | ** | |

EC: Electrical conductivity, CEC: Cation exchange capacity, BET: Specific surface area.

Values followed by different lowercase letters within a column represent significant differences among the treatments in the same feedstock at P<0.05. F: Feedstock; T: Temperature; F×T: Interaction between feedstock and temperature; * and ** represent a significant difference at P<0.05 and P<0.01, respectively; ns represents a non-significant difference.

**Fig.S1** TG images of different temperature of the (a) RS, (b) CS, (c) OT and (d) GW

**Fig.S2** N_2_ adsorption-desorption isotherms of GW3, GW4, GW5, GW6, and GW7

**Fig.S3** FTIR spectra of (a) RS, (b) CS, (c) OT and (d) GW biochars prepared at 300℃ to 700℃






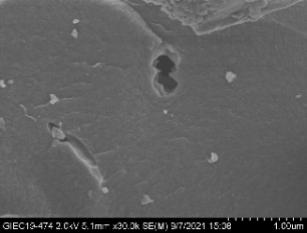

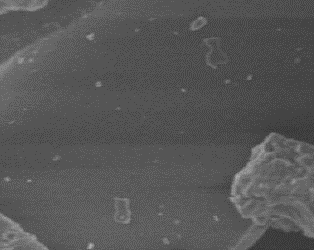






1μm

1μm


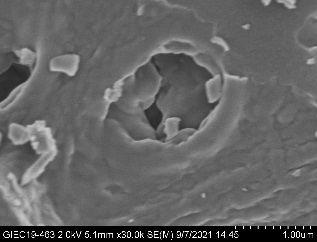


RS6

CS6


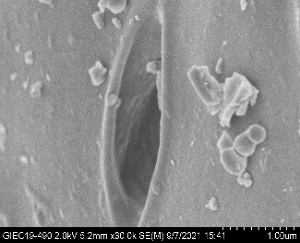


OT6

**(e)**

GW6

1μm

1μm




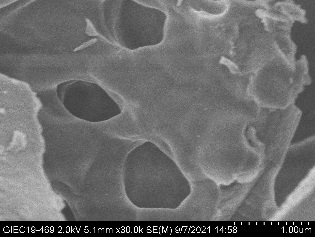



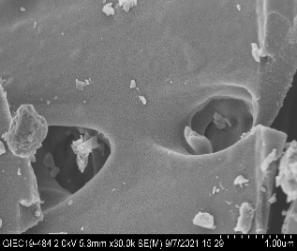


1μm

1μm

1μm

1μm

RS7

OT7

GW7

**(f)**

CS7


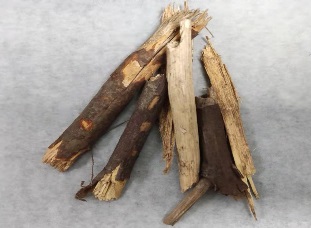

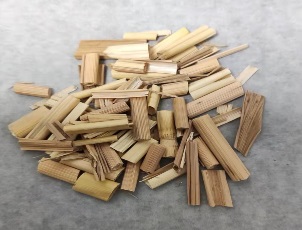

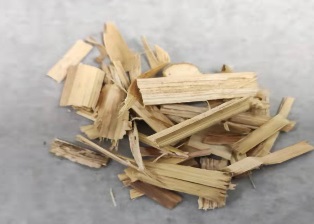

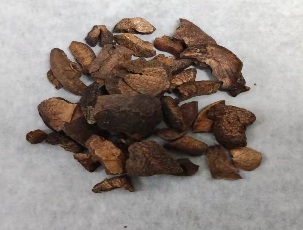


**Rice straw**

**Corn straw**

**Camellia oleifera shells**

**Garden waste**

**(a)**







1μm

1μm


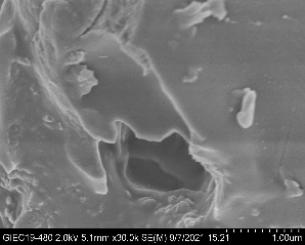


RS3

CS3

OT3

**(b)**

GW3


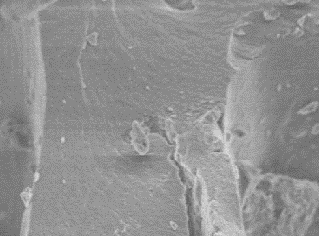


1μm

1μm

OT3


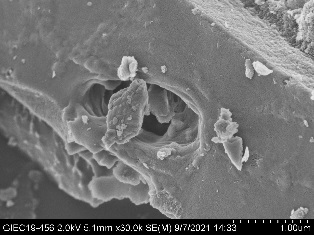





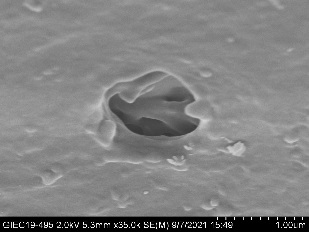


RS4

CS4

RS5

CS5

**(d)**

RS4

CS4

**(c)**

**(c)**

1μm

1μm

1μm

1μm

1μm

1μm

GW5

OT4

OT5

GW5

OT4

GW4

1μm

1μm

1μm

1μm

1μm

1μm

**Fig.S4** The photos of woody plants and herbaceous plants raw materials (a), SEM images of RS, CS, OT and GW prepared at (b) 300, (c) 400, (d) 500, (e) 600, (f) 700℃, respectively
